# Phenylboronic Acid-Functionalized Layer-by-Layer Assemblies for Biomedical Applications

**DOI:** 10.3390/polym9060202

**Published:** 2017-05-31

**Authors:** Baozhen Wang, Kentaro Yoshida, Katsuhiko Sato, Jun-ichi Anzai

**Affiliations:** 1 Department of Nutrition and Food Hygiene, School of Public Health, Shandong University, 44 Wenhua Xilu, Jinan 250012, China; bzhenw@hotmail.com; 2 School of Pharmaceutical Science, Ohu University, 31-1 Misumido, Tomita-machi, Koriyama, Fukushima 963-8611, Japan; k-yoshida@pha.ohu-u.ac.jp; 3 Graduate School of Pharmaceutical Sciences, Tohoku University, Aramaki, Aoba-ku, Sendai 980-8578, Japan; satok@m.tohoku.ac.jp

**Keywords:** phenylboronic acid, layer-by-layer, biosensors, drug delivery systems

## Abstract

Recent progress in the development of phenylboronic acid (PBA)-functionalized layer-by-layer (LbL) assemblies and their biomedical applications was reviewed. Stimuli-sensitive LbL films and microcapsules that exhibit permeability changes or decompose in response to sugars and hydrogen peroxide (H_2_O_2_) have been developed using PBA-bearing polymers. The responses of PBA-modified LbL assemblies arise from the competitive binding of sugars to PBA in the films or oxidative decomposition of PBA by H_2_O_2_. Electrochemical glucose sensors have been fabricated by coating the surfaces of electrodes by PBA-modified LbL films, while colorimetric and fluorescence sensors can be prepared by modifying LbL films with boronic acid-modified dyes. In addition, PBA-modified LbL films and microcapsules have successfully been used in the construction of drug delivery systems (DDS). Among them, much effort has been devoted to the glucose-triggered insulin delivery systems, which are constructed by encapsulating insulin in PBA-modified LbL films and microcapsules. Insulin is released from the PBA-modified LbL assemblies upon the addition of glucose resulting from changes in the permeability of the films or decomposition of the film entity. Research into insulin DDS is currently focused on the development of high-performance devices that release insulin in response to diabetic levels of glucose (>10 mM) but remain stable at normal levels (~5 mM) under physiological conditions.

## 1. Introduction

**Figure 1:**
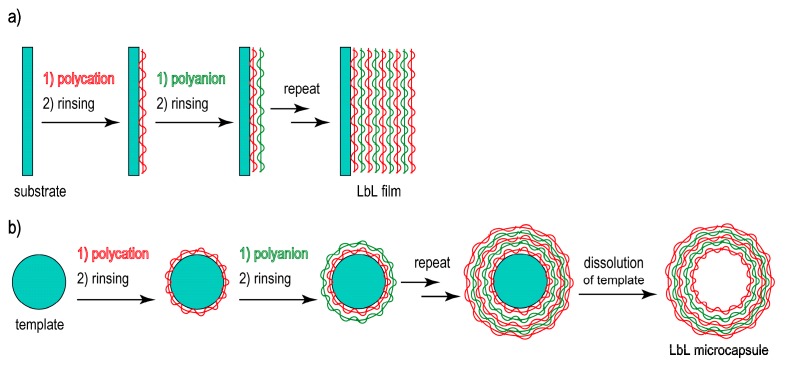
Preparation of layer-by-layer (LbL) films (**a**) and microcapsules (**b**).

Multilayer thin films are prepared by a layer-by-layer (LbL) deposition of polymeric materials on the surface of a solid substrate through attractive forces including electrostatic interactions, hydrogen bonds, covalent bonds, molecular recognitions, and biological affinity [1,2,3,4,5]. Thus, a variety of synthetic and biological materials can be used as components of LbL thin films. LbL films can be deposited on a variety of solid substrates, including metals, glass, polymers, organic crystals, and biological samples such as cells and tissues. LbL films were first prepared in the early 1990s by the alternate deposition of synthetic polymers through electrostatic affinity [6,7]. The adsorption of polyelectrolytes on a charged surface produces a new surface with the opposite charge, resulting from overcompensation of the surface charge. The over-compensated surface enables deposition of the next layer of an oppositely charged polymer. In a typical procedure, the solid substrate is alternately immersed in aqueous solutions of the polymers for 15–30 min to deposit them on the surface of the substrate, followed by rinsing to remove nonspecifically or weakly adsorbed polymers (Figure 1a). It is a merit of the LbL deposition technique that the thickness of the films can be regulated simply by changing the number of deposited layers, because film thickness increases with the number of depositions. In addition to synthetic polymers, biopolymers such as proteins [8,9], polysaccharides [10,11], and DNA [12,13] are also used as building blocks for the construction of LbL films, because these biopolymers contain net electrical charges. The thicknesses of LbL films composed of polysaccharides or polypeptides often increase exponentially with the number of layers [14,15], in contrast to the linear growth of LbL films consisting of synthetic polymers.

Hydrogen bonding and covalent bonding are also feasible driving forces for the construction of LbL films. Poly(carboxylic acid)s are often used to construct hydrogen bond-based LbL films by combining poly(ethyleneglycol) and poly(vinylpyrrolidone) as the hydrogen bonding acceptors [16,17]. An interesting feature of hydrogen-bonded LbL films is their pH-dependent stability. LbL films consisting of poly(carboxylic acid)s are stable in acidic media, whereas they decompose at neutral and basic pH because the hydrogen bonds are broken as a result of deprotonation of the carboxylic acid residues. The pH-sensitive nature of hydrogen-bonded LbL films can be exploited in pH-triggered release devices [18]. On the other hand, covalently bonded LbL films have been prepared using click reaction of divalent triazolinedione and trivalent diene and aminolysis reactions of dimethylazlactone polymers [19,20]. Thiol-bearing polymers are used to stabilize LbL films through covalent crosslinking of prepared LbL films [21].

Host-guest interactions and biological affinities are also available as driving forces for the construction of LbL films. Cyclodextrin (CD)-bearing polymers serve as host polymers in LbL films, in which guest compounds can be included in the CD cavity. Adamantane [22], azobenzene [23], and ferrocene [24] are frequently used as guest molecules in CD polymer-based LbL films. Proteins with multiple binding sites, in which protein-ligand interactions drive the formation of the film, are exploited for the construction of LbL films. Examples include concanavalin A (Con A) [25] and avidin [26], which specifically bind sugar and biotin, respectively. Therefore, sugar- and biotin-labeled polymers and biomolecules can be built into LbL films. Interestingly, glycoenzymes equipped with intrinsic hydrocarbon chains, such as glucose oxidase (GOx) [27] and horseradish peroxidase (HRP) [28], can be used as film components without labeling in the Con A-based protocol.

Hollow microcapsules can be constructed by LbL deposition of polymers on the surfaces of colloidal particles, followed by dissolution of the core (Figure 1b) [29]. CaCO_3_ particles are often used for encapsulating proteins and drugs in microcapsules because the CaCO_3_ template can be easily dissolved in acidic solutions or aqueous ethylenediamine-*N*,*N*,*N′*,*N′*-tetraacetic acid (EDTA) solutions. Polymer microcapsules thus prepared are stable over a wide pH range and permeability can be manipulated by changing the environmental pH [30]. Therefore, LbL microcapsules are attracting much attention in the development of drug delivery systems [31]. An advantage of LbL microcapsules is that the structure of their shell membrane can be tailored at the molecular level by suitable building block materials. Thus, LbL films and microcapsules have applications in a variety of devices, including separation and purification membranes [32], optical and electrochemical sensors [33,34], and controlled release systems [35,36].

**Figure 2:**
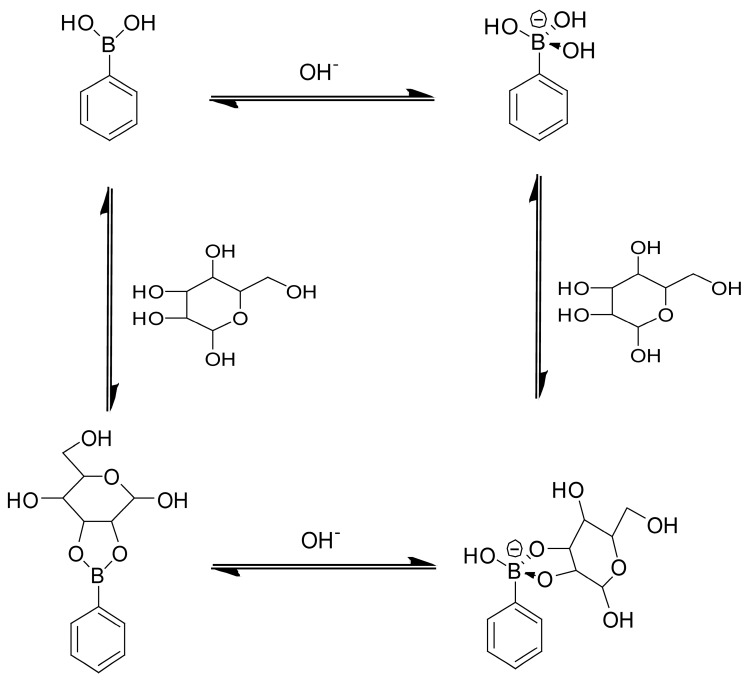
Binding equilibria of PBA with sugar.

**Figure 3:**
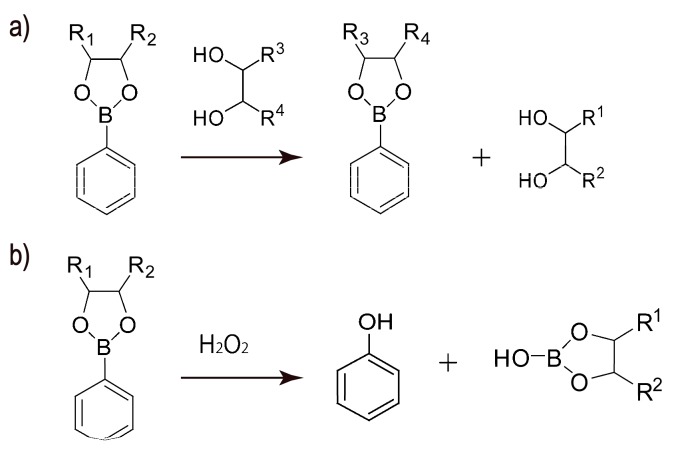
(**a**) Competitive binding of a diol compound to a boronate ester; and (**b**) decomposition of boronate ester by H_2_O_2_.

Recently, phenylboronic acid (PBA) and its derivatives have attracted much attention as modifiers of LbL films and microcapsules because they selectively bind 1,2- and 1,3-diol compounds, such as sugars. Figure 2 shows the binding equilibrium of neutral and negatively charged forms of PBA with sugar. PBAs are in a trigonal planar molecular geometry in acidic media. In contrast, in basic media, PBAs assume charged tetragonal form as a result of the addition of –OH^−^ ion to the boron atom. Both neutral and charged PBAs bind sugars to form boronate esters. Note that boronate esters can be negatively charged even in neutral solutions, in which the parent PBA is in a neutral form, through the addition of –OH^−^ ions, because the p*K*_a_ values of boronate esters are usually lower than those of the parent PBA compounds [37,38]. That is, the electronic state of PBA compounds changes upon binding to sugars, resulting in significant changes in the optical and electrochemical properties of the PBAs. Consequently, many PBA derivatives have so far been synthesized to develop optical and electrochemical sensors [39,40,41,42,43,44,45,46,47,48]. Another interesting feature of PBA esters relates to the reversibility of the formation of their ester bonds. That is, boronate esters easily decompose into PBA and diol compounds upon acidification because of the unstable nature of boronate esters in the neutral form compared to negatively charged esters [37,38]. Furthermore, diol moieties in PBA esters can be replaced by different diols through competitive binding in the presence of excess amounts of different diols under mild conditions, such as in neutral aqueous media at ambient temperature (Figure 3a). In addition, PBAs and their esters are sensitive to reactive oxygen species, such as hydrogen peroxide (H_2_O_2_). For example, PBAs and their esters irreversibly decompose resulting from the oxidative scission of carbon-boron bonds in PBA and esters by H_2_O_2_, as shown in Figure 3b [39,40]. The stimuli-sensitive nature of PBAs and their esters makes them suitable modifiers of LbL films and microcapsules for the construction of biosensors and stimuli-sensitive systems. In fact, PBA-modified LbL films and microcapsules have been extensively studied for the past several years. Therefore, this review focuses on the construction of PBA-functionalized LbL films and microcapsules and their applications to the development of stimuli-sensitive systems, biosensors and bio-interfaces, and drug delivery systems.

## 2. Stimuli-Sensitive LbL Assemblies

**Figure 4:**
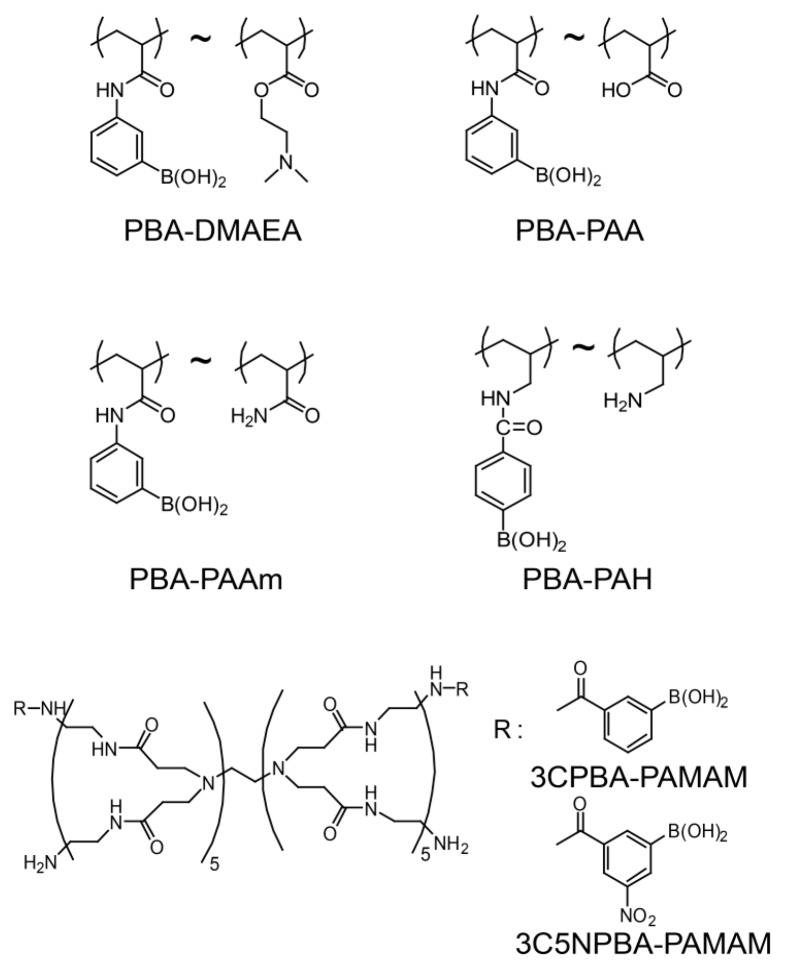
Chemical structures of phenylboronic acid (PBA)-modified polymers.

Stimuli-sensitive LbL films and microcapsules that decompose in response to pH changes, sugars, and H_2_O_2_ have been constructed using PBA-modified polymers as the film component. De Smedt and coworkers prepared LbL films by alternate deposition of a poly(acrylic acid) (PAA) derivative with pendant PBA groups (PBA-DMAEA polymer) (Figure 4) and poly(styrene sulfonate) (PSS) on the surface of microbeads through the electrostatic affinity between the polymers. LbL microcapsules with PBA-DMAEA/PSS shell have also been prepared by dissolving the microbead cores [49]. The LbL microcapsule is stable in the buffer solution at pH 9.0 and completely decomposes in the presence of 5 mg·mL^−1^ glucose after 5 min. To our knowledge, this is the first report on the glucose-induced decomposition of PBA-based LbL films and microcapsules. The results were rationalized based on the excess negative charges along the PBA-DMAEA polymer chains in the microcapsule’s shell, induced by glucose binding to the PBA moieties. One problem is that this microcapsule does not decompose at physiological pH, probably due to the low affinity of glucose to the PBA moiety. The use of PBA derivatives with higher affinity to glucose at physiological pH may improve the responses of the microcapsules.

Another mechanism in sugar-induced decomposition of PBA-based LbL assemblies was provided by Levy and coworkers [50]. They used PBA-modified PAA (PBA-PAA, Figure 4) and polysaccharide mannan to construct LbL films and microcapsules, in which LbL layers were assembled by forming boronate ester bonds between the PBA residues of PBA-PAA and 1,2-diol units in mannan. The PBA-PAA/mannan LbL film can be constructed at pH 9 or higher, whereas film production is unsuccessful at pH 8 and 7 due to the low affinity of PBA-PAA to mannan. PBA-PAA/mannan LbL films prepared at pH 11 are stable in buffer solution at pH 9, while they decompose upon immersion in pH 8 solution within 1 min. Thus, PBA-PAA/mannan LbL films are sensitive to pH changes. Furthermore, PBA-PAA/mannan LbL films decompose in response to sugars, such as fructose, glucose, mannose, and galactose at the concentration range 5 × 10^−3^–2.5 × 10^−2^ M. This is because the boronate ester bonds in the LbL films are cleaved in the sugar solutions as a consequence of competitive binding of sugars to PBA-PAA. Note that the mechanism for sugar-induced decomposition of the PBA-PAA/mannan films differs from the decomposition mechanism in PBA-DMAEA/PSS films reported by De Smedt and coworkers. Microcapsules have also been successfully prepared by the deposition of PBA-PAA/mannan film on the surface of CaCO_3_ microspheres followed by dissolution of the CaCO_3_ core. The microcapsules decompose in 1 × 10^−3^–3 × 10^−3^ M fructose solution and 1 × 10^−2^–3 × 10^−2^ M glucose, mannose, and galactose solutions at pH 11. However, the sugar response of the PBA-PAA/mannan LbL films and microcapsules could not be studied under physiological conditions owing to their instability at neutral pH. Thus, these studies demonstrated that PBA-modified polymers are promising for the construction of sugar-sensitive LbL films and microcapsules, although the response to glucose under physiological conditions must be improved for biomedical applications of LbL assemblies.

In order to improve the responses of PBA-based LbL films under physiological conditions, several groups, including our group, have constructed PBA-based LbL films using different materials. Zhang and coworkers prepared LbL films composed of poly(acrylamide) copolymer bearing PBA side chains (PBA-PAAm, Figure 4) and poly(vinyl alcohol) (PVA), in which the PBA moieties of PBA-PAAm formed boronate ester bonds with 1,3-diol units of PVA [51]. The 9-bilayer (PBA-PAAm/PVA)_9_ film shows glucose sensitivity in buffer solutions at pH 7.5 and 8.5 in the presence of 5–30 mM glucose. The (PBA-PAAm/PVA)_9_ film decomposes by about 25% after 200 h in 15 mM glucose solution at pH 7.5, although it is slightly unstable even in the absence of glucose. The response of this film is rather slow, compared to the rapid response of LbL assemblies based on PBA-DMAEA/PSS [49] and PBA-PAA/mannan systems [50]. Therefore, (PBA-PAAm/PVA)_9_ films may be useful for the sustained release of insulin. Another group used PBA-PAAm combined with mucin, a glycoprotein found in the mucosal surfaces of animal epithelial tissues, as a counter material for the construction of LbL films [52]. The (PBA-PAAm/mucin)_10_ film is stable in buffer solutions at pH 7.4 and 9.0. However, it slightly decomposes upon the addition of 5 mg·mL^−1^ (ca. 28 mM) glucose. In other works, PBA-modified chitosan and poly(vinylamine) have been used to construct LbL films, although the responses of the films to external stimuli were not studied [53,54].

**Figure 5:**
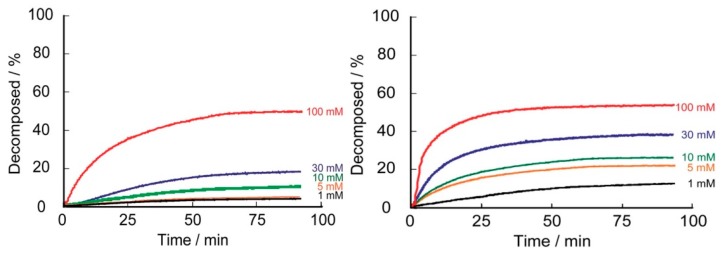
Glucose-induced decomposition of (PVA/3CPBA-PAMAM)_10_ (**left**) and (PVA/3C5NPBA-PAMAM)_10_ (**right**) films in 1–100 mM glucose solutions at pH 7.4 and 37 °C. Reprinted with permission from Ref. [55]. Copyright 2014 Royal Society of Chemistry.

We have recently used PBA-modified dendrimers to further improve pH stability and the sugar-responses of LbL films [55,56,57,58,59]. To achieve this goal, fourth-generation poly(amidoamine) (PAMAM) dendrimers were covalently modified with PBA derivatives substituted with nitro groups (Figure 4). It was anticipated that the p*K*_a_ of the nitro group-substituted 3C5NPBA residue in the dendrimers would be lower than that of unsubstituted 3CPBA due to the electron-withdrawing effect of the nitro group. In fact, the p*K*_a_ of 3-nitro-PBA is reported to be 7.1, compared with 8.8 for that of unsubstituted PBA [38]. Thus, LbL films consisting of 3C5NPBA-PAMAM may exhibit improved responses to glucose at physiological pH. Furthermore, we expected that dendrimer-based LbL films would show rapid response to glucose because the number of PBA residues in the PAMAM dendrimer is limited. That is, the number of boronate ester bonds between the single dendrimer molecule and PVA should be limited in the film. Figure 5 shows the kinetics of the glucose-induced decomposition of (PVA/3CPBA-PAMAM)_10_ and (PVA/3C5NPBA-PAMAM)_10_ films. The responses of LbL film prepared using 3C5NPBA-PAMAM are rapid and significantly enhanced, compared to unsubstituted PBA films. Thus, the use of substituted PBA is a promising strategy for improving the responses of PBA-modified LbL films. A variety of PBA derivatives with different substituents are commercially available.

**Figure 6:**
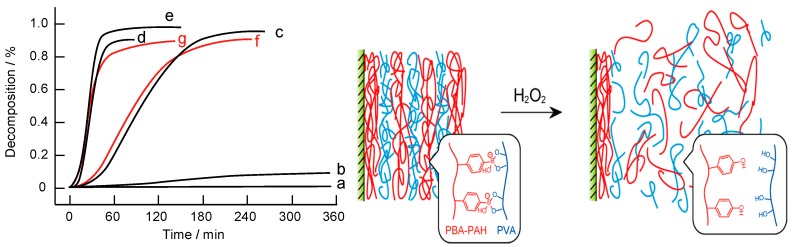
(**left**) Decomposition of (PBA-PAH/PVA)_10_ film in the presence of H_2_O_2_ at pH 7.4 (a–e), pH 6.0 (f), and pH 9.0 (g). H_2_O_2_ concentrations were 0 (a), 0.05 (b), 0.1 (c), 0.5 (d), and 1.0 mM (e–g); (**right**) schematic illustration of the H_2_O_2_-induced decomposition of (PBA-PAH/PVA)_10_ LbL film. Reprinted with permission from Ref. [60]. Copyright 2014 American Chemical Society.

PBA and its boronate esters are sensitive to reactive oxygen species (ROS), such as H_2_O_2_, thus providing an opportunity to develop ROS-sensitive LbL assemblies by using PBA-modified polymers. As described in Section 1 (Figure 3b), carbon-boron bonds in PBA and boronate esters are oxidatively cleaved by H_2_O_2_, which enables PBA-based LbL assemblies to decompose in response to H_2_O_2_. According to this strategy, we have prepared LbL films composed of PBA-modified poly(allylamine) (PBA-PAH, Figure 4) and PVA to evaluate the response to H_2_O_2_ [60]. Figure 6 shows the decomposition kinetics of (PBA-PAH/PVA)_10_ film in 0–1.0 mM H_2_O_2_ solutions. As expected, the LbL film decomposes in the presence of H_2_O_2_, the degree of decomposition being dependent on H_2_O_2_ concentration. H_2_O_2_-induced film decomposition can be observed at pH 6–9. The decomposition of the LbL film is schematically illustrated in Figure 6 (right).

As an extension of the H_2_O_2_-sensitive system, (PBA-PAH/PVA)_10_ film has been combined with GOx to prepare glucose-sensitive LbL films [61]. The GOx(PBA-PAH/PVA)_10_ film fully decomposes within 120 and 60 min in 1.0 and 10 mM glucose solutions, respectively, at pH 7.4 resulting from the enzymatic production of H_2_O_2_ through GOx-catalyzed oxidation reaction of glucose (Equation (1)). On the other hand, only 9% and 15% of film decomposition was observed in 1 mM mannose and fructose solutions at pH 7.4, respectively. Thus, this GOx-aided system shows high selectivity to glucose. It is noteworthy that (PBA-PAH/PVA)_10_ film is highly stable in glucose-free solutions at physiological pH because it is prepared using PBA-PAH containing a large amounts of PBA residues (i.e., 26%), while the film almost fully decomposes in response to physiologically relevant levels of glucose due to irreversible scission of the carbon-boron bonds. H_2_O_2_-sensitive LbL films could be combined with a variety of enzymes that produce H_2_O_2_ to construct stimuli-sensitive systems.

Glucose + O_2_ → Gluconolactone + H_2_O_2_ (1)

## 3. Biosensors and Biointerfaces

Early works demonstrated that LbL films are applicable to the fabrication of electrochemical enzyme biosensors, in which the surface of the electrode is coated with enzyme LbL films [62,63,64,65,66]. The advantages of LbL films in biosensor constructions include the facile preparation of thin layers with defined thickness, precise control of the internal structure of the films, and possible use of proteins and redox mediators as components of the LbL films. These advantages enable optimization of the performance characteristics of biosensors by tailoring the LbL films at the molecular level. For instance, Yang and coworkers have constructed H_2_O_2_ biosensors by depositing LbL films consisting of PBA-coated Au nanoparticles (PBA-NPs) and PVA on the surface of indium tin oxide (ITO) electrode. The surface of the LbL film was subsequently modified with HRP [67]. The H_2_O_2_ sensor thus prepared shows a voltammetric response to H_2_O_2_ in the concentration range 2.8 × 10^−5^–7.3 × 10^−3^ M.

In other studies, LbL films consisting of PBA-bearing polymers coupled with GOx have been utilized for the construction of glucose biosensors, in which LbL films were deposited on a pyrolytic graphite (PG) electrode via boronate ester bonds between the polymer and hydrocarbon chains of GOx [68]. The GOx-modified PG electrode thus prepared shows a voltammetric response to glucose, confirming that GOx retains its catalytic activity in the LbL film. It has also been possible to use a copolymer consisting of PBA and ferrocene (Fc) monomer units (PBA-Fc copolymer) and PVA-modified GOx for the preparation of glucose biosensors [69]. The PBA-Fc copolymer and PVA-modified GOx are spin-coated in a layer-by-layer fashion on the electrode to form a thin film. The redox current of the sensors originating from Fc moiety increases with increasing numbers of LbL layers, as in the case of other Fc-containing LbL films [70,71]. The output current of the sensors depends on the number of GOx layers, confirming the effective electron transfer between GOx and the Au electrode mediated by Fc residues. These works demonstrated the usefulness of PBA-modified polymers in the construction of enzyme biosensors. However, it should be noted that boronate ester bonds are cleaved by H_2_O_2_, as discussed in the previous section. Therefore, both the reusability and stability of enzyme sensors should be carefully evaluated if H_2_O_2_ is involved in the enzymatic reactions.

**Figure 7:**
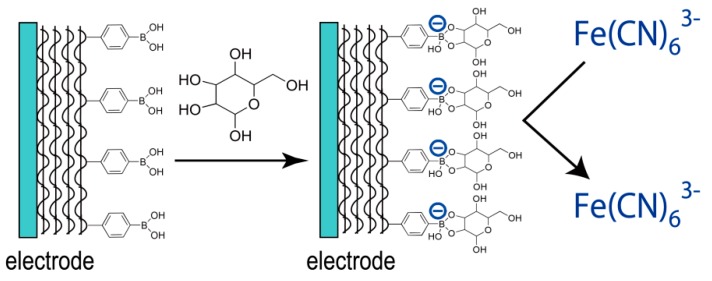
A schematic illustration of the response mechanism of PBA film-coated electrode sensitive to sugars.

PBA derivatives have widely been utilized in the construction of non-enzymatic glucose sensors because of their high affinity for sugars [72,73,74,75,76]. The binding affinity of PBAs for glucose is rather low compared with that for other sugars such as fructose and mannose [38]. However, PBAs are still useful for the construction of non-enzymatic glucose sensors because these sensors are usually used for detecting glucose at millimolar levels in samples such as human blood, which contains a lower level of other sugars. One problem in the construction of PBA-based non-enzymatic biosensors in electrochemical detection mode is that no electrical signal can be obtained from the binding of glucose to PBAs. This is because the formation of the glucose-PBA adduct does not produce redox active products, unlike enzymatic reactions. Therefore, PBA-based non-enzymatic glucose sensors must be coupled with redox-active compounds to obtain output signals. In fact, Au disk electrodes coated with LbL films composed of PBA-PAH have been studied as non-enzymatic glucose sensors in combination with ferricyanide ions, Fe(CN)_6_^3−^, as a redox marker [77]. The PBA-PAH film-coated Au electrodes exhibit voltammetric response to Fe(CN)_6_^3−^ ions in the absence of glucose, while the response is suppressed upon the addition of glucose into the sample solution, resulting from the binding of glucose to PBA-PAH in the film. Figure 7 illustrates the mechanism by which the voltammetric response of the sensor is suppressed in the presence of glucose. Reagentless glucose sensors that do not require soluble redox markers could be developed if LbL films were prepared using redox-active PBAs [78,79].

**Figure 8:**
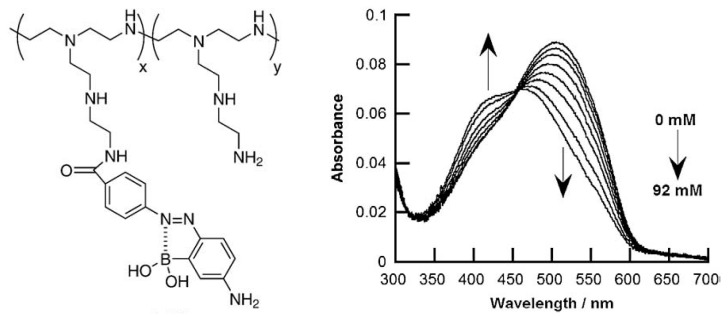
(**left**) Chemical structure of PEI modified with boronic acid-substituted azobenzene dye; (**right**) Absorption spectra of the azobenzene-modified PEI in the absence and presence of glucose. Reprinted with permission from Ref. [80]. Copyright 2009 from Elsevier.

In colorimetric and fluorometric glucose sensors, boronic acid-substituted dyes can be used as glucose-recognition elements. For example, LbL films have been prepared using poly(ethyleneimine) (PEI) modified with boronic acid-substituted azobenzene dye, which exhibits changes in color upon glucose binding (Figure 8) [80]. Three types of LbL films of the azobenzene-modified PEI have been prepared using poly(vinyl sulfate) (PVS), carboxymethylcellulose (CMC), and alginic acid (AGA) as the anionic counterparts. PVS- and CMC-based films show an absorption maximum at 500 nm and the absorption band is blue-shifted upon adding 1–92 mM glucose to the solution. In contrast, the AGA-based film shows an absorption maximum at 460 nm even in the absence of glucose, and no spectral change is observed upon the addition of glucose. The results were rationalized based on the fact that AGA forms ester bonds with the azobenzene dyes in the film, which excludes the binding of glucose to the dye. Thus, careful attention should be paid to the type of polysaccharides used for the preparation of PBA-containing LbL films [57].

It is not essential to use boronic acid-substituted dyes for the construction of optical sensors. Instead, anionic dyes have been immobilized electrostatically to PBA-containing LbL films for the development of colorimetric sensors for fructose and glucose [81,82]. The dye-immobilized LbL films exhibit changes in color upon exposing the films to sugar solutions. The colorimetric response of the films was ascribed to the release of dye from the LbL film, arising from the generation of negative charges in the film on the binding of sugars to the PBA residues. It is a merit of LbL films used as scaffolds for the immobilization of anionic dyes that the response is rapid compared with the slow responses of bulk polymer films. Boric acid (H_3_BO_3_) has also been used in combination with the fluorescent dye alizarin red S (ARS) for the construction of fluorescence sensors based on LbL films [83]. ARS-H_3_BO_3_ adducts were built into LbL films together with layered double hydroxides (LDHs) to study the fluorescence properties in the presence of Cu^2+^ ion and tiopronin (a clinical drug for cystinuria). The LbL films of the ARS-H_3_BO_3_ adduct emit strong fluorescence, which is quenched by the Cu^2+^ ion through the formation of ARS-Cu^2+^ complexes. Conversely, upon the addition of tiopronin, the fluorescence of ARS is restored through competitive binding of tiopronin to Cu^2+^ ion. Thus, LbL films of the ARS-H_3_BO_3_ adduct could detect tiopronin in the concentration range of 0–8 × 10^−^^8^ g·mL^−1^.

An optical sensor for glucose has been developed using PAAm-PBA/PVA films (Figure 4), in which glucose-induced changes in film thickness are monitored by an optical fiber probe [84]. LbL films with 30–90 bilayers were used to monitor glucose-induced changes in thickness. Film thicknesses change depending on the linear concentration of glucose in the range of 0–10 mM. The response time of the films is approximately 10 min and changes in thickness are fully reversible. The response was ascribed to the swelling and deswelling of the film resulting from enhanced negative charges in the film, which in turn originated from glucose binding to the PBA moieties in the film.

LbL films consisting of PBA-modified polymers have been used in the construction of bio-interfaces for cell patterning [85,86,87,88]. Multilayered gel films are prepared through the dropping/spinning-aided LbL protocol using a copolymer bearing PBA and phosphorylcholine groups. Interestingly, spatially defined cell-laden layers are prepared by depositing cell dispersion on the gel films, thus enabling the potential use of the LbL gel films for the study of distant-dependent cell-cell interactions. LbL depositions of poly(amidoamine) linear polymers with PBA pendant groups and PVA or chondroitin sulfate afford biointerfaces for cell attachment and proliferation [89]. The LbL films provide suitable bio-interfaces for cell attachment and the cells retain metabolic activity, therefore confirming the biocompatibility of the films.

## 4. Drug Delivery Systems

**Figure 9:**
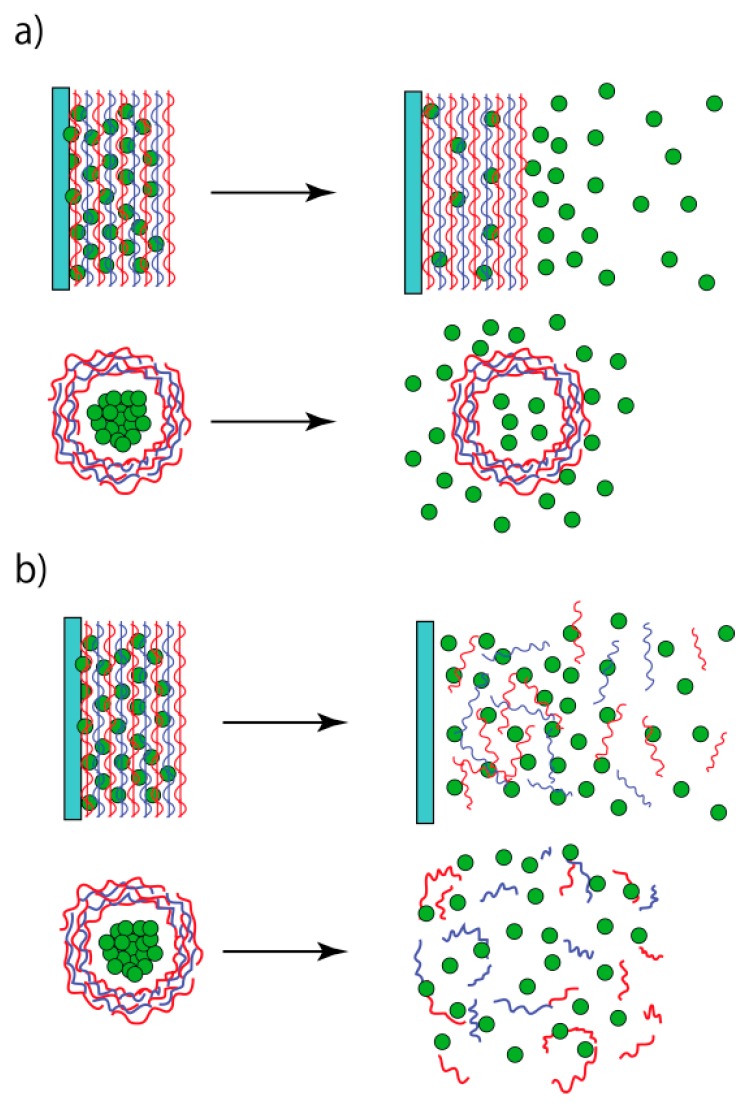
Drug release from LbL films and microcapsules through (**a**) permeability changes and (**b**) decomposition.

LbL films and microcapsules are suitable carriers of drugs for controlled delivery [90,91,92,93,94,95,96]. The advantages of LbL films and microcapsules as drug carriers include facile preparation under mild conditions, precise control of film thickness by changing the number of layers, and a wide choice of materials such as biocompatible and biodegradable polymers. Two different routes are available for controlled drug release from PBA-modified LbL films and microcapsules, as schematically illustrated in Figure 9. An embedded drug can be released from LbL films and microcapsules through permeability changes in the LbL layer (Figure 9a), which may result from an increased number of negatively charged boronate esters or a decreased number of crosslinks in the films. In extreme cases, LbL films and microcapsules may be decomposed (Figure 9b). In the former mechanism, pulsed release systems, in which drug release is accelerated and suppressed alternately in an on-off fashion in response to external stimuli, may be constructed. On the other hand, decomposition of the film entity would result in a burst release of drugs. It is anticipated that permeability changes or decomposition of PBA-modified LbL films and microcapsules can be triggered by various stimuli, as discussed in the previous section.

Glucose-sensitive LbL films and microcapsules are of special interest because of their potential use in insulin delivery systems for the treatment of diabetic patients. Therefore, a variety of LbL assemblies have been constructed for insulin delivery using glucose-sensitive materials such as enzymes and lectins [97,98,99]. Enzyme- and lectin-based systems show high selectivity to glucose owing to the high specificity of the proteins. However, in some cases, contamination of the proteins may be problematic in the practical use of such systems. Therefore, insulin delivery systems based on synthetic materials are highly desirable. In this context, several groups have reported PBA-containing LbL films and microcapsules for insulin delivery. Our group prepared insulin-embedded microcapsules by LbL deposition of PBA-PAH and AGA on the surface of insulin-containing CaCO_3_ microspheres, followed by dissolution of the core CaCO_3_ [100]. The microcapsules release insulin into buffer solutions at pH 9.0 upon the addition of 10–100 mM glucose. The sugar-triggered release of insulin was ascribed to the decomposition of the microcapsules. Unfortunately, the response of the microcapsules to glucose is not satisfactory at physiological pH. Recently, two groups have reported the preparation of polysaccharide-based LbL microcapsules for the controlled release of insulin. Insulin-loaded LbL microcapsules are prepared by LbL deposition of PBA-bearing poly(acrylate) and polysaccharide chitosan on the surface of insulin-adsorbed silica (SiO_2_) microspheres and subsequent dissolution of the SiO_2_ core in NH_4_F/HF buffer solution [101]. A similar LbL protocol using PBA-bearing chitosan and AGA also provides insulin-loaded LbL microcapsules [102]. Notably, these polysaccharide-based microcapsules exhibit low cytotoxicity owing to the biocompatibility of polysaccharides.

PBA-modified thin films have been used for the construction of scaffolds for glucose-induced insulin release [103]. To achieve this, PVA-tagged insulin has been built into LbL films, in which PVA-tagged insulin forms boronate esters with PAAm-PBA. The release of insulin from the LbL films is accelerated by glucose to some extent at pH 8.0 and 9.0, while at pH 7.4 the effect of glucose is limited. It is desirable that insulin-loaded LbL films and microcapsules release insulin in response to diabetic levels of glucose (>10 mM) and remain stable at normal levels (~5 mM) under physiological conditions. Therefore, further improvement in the stability and response to glucose is required for these LbL films and microcapsules.

The controlled release of small molecules, such as ARS [104], doxorubicin (DOX) [105,106], and paclitaxel (PTX) [107,108], from LbL assemblies has also been studied. In these studies, the small molecules were encapsulated in LbL films through boronate ester bonds [104], physical adsorption [105], covalent bonds [106], and hydrophobic interactions [107,108]. Interestingly, LbL films consisting of DOX-bearing polymers and PVA show a zero-order release of DOX when the films are prepared using PVA with a narrow molecular weight distribution [106]. PTX-loaded films are able to regulate cell proliferation through controlled release of PTX, arising from precise control of the location of PTX in the films [108].

## 5. Conclusions

PBA-functionalized LbL films and microcapsules have widely been studied for the development of glucose-sensitive devices, such as glucose sensors and drug delivery systems. One of the challenges of PBA-based glucose sensors is to develop reagentless sensors that can be used without the addition of redox markers in sample solutions. To achieve this goal, redox markers have to be immobilized on the surface of the electrode together with PBA. In drug delivery systems, glucose-triggered delivery devices that release insulin only when the blood glucose is higher than the diabetic level (>10 mM) under physiological conditions are highly desirable. PBA-functionalized LbL films and microcapsules would be useful for constructing such devices because PBA derivatives exhibit low toxicity and low immunogenicity. These devices would be realized by improving the glucose-binding ability of PBAs and the suitable design of the chemical structures of PBA-polymers. Furthermore, a novel strategy for constructing LbL assemblies would contribute to the development of high-performance sensors and drug delivery systems.
